# A Predictive Model for Nursing Students’ Person-Centered Care Competency: Focusing on Patients with Dementia

**DOI:** 10.3390/healthcare14121683

**Published:** 2026-06-12

**Authors:** So-Hee Lim

**Affiliations:** Department of Nursing, Kyungmin University, Uijeongbu-si 11618, Republic of Korea; sweetnurseme@naver.com; Tel.: +82-31-828-7472

**Keywords:** nursing student, person-centered care, empathy, clinical practice adaptation, nursing professionalism

## Abstract

**Background/Objectives**: This study aimed to verify a prediction model identifying the relationships and pathways among factors associated with Korean nursing students’ provision of person-centered care to patients with dementia. **Methods**: This was a covariance structure analysis study to establish a hypothetical model of 313 Korean nursing students located in a metropolitan area. IBM SPSS version 18.0 (Chicago, IL, USA) and AMOS version 5.0 (Chicago, IL, USA) were used to analyze the data. Structural equation modeling analysis was applied to verify convergent and discriminant validity using higher-order factor analysis in the final model analysis. **Results**: The model fit indices of the research model were as follows: χ^2^/df = 1.83 (*p* < 0.001), GFI = 0.91, AGFI = 0.88, NFI = 0.91, CFI = 0.90, RMR = 0.04, and RMSEA = 0.05. The factors affecting person-centered care, nursing professionalism (γ = 0.45, *p* = 0.024), and empathy (γ = 0.21, *p* = 0.036) showed significant associations, whereas clinical practice adaptation (γ = 0.21, *p* = 0.013) and nursing professionalism (γ = 0.08, *p* = 0.004) had indirect effects. These factors explained 40% of the variance in person-centered care. **Conclusions**: This study is significant because it provides basic data for developing an educational program that can improve the person-centered care capacity of domestic nursing students by confirming that clinical practice adaptation, nursing professionalism, and empathy are important factors related to person-centered care.

## 1. Introduction

As life expectancy increases with the development of advanced medical technology, South Korea is becoming a rapidly aging country. Those aged 65 years or older accounted for 15.7% of the total South Korean population in 2020 and are expected to account for 45.9% in 2060 and 46.1% in 2065, creating a super-aged society [1,2]. As South Korea progresses toward becoming a super-aged society, geriatric diseases are increasing in prevalence. The number of patients with dementia was approximately 860,000 in 2020 and is expected to exceed 1.36 million in 2030, 2.2 million in 2040, and 3 million in 2050 [2].

Dementia refers to a group of various symptoms related to a decline of cognitive function and cases in which cognitive dysfunction, including memory impairment without impaired consciousness, appears continuously owing to brain disease or damage [3]. Dementia is a degenerative neurological disease that progresses slowly over time, resulting in cognitive decline and behavioral disorders that make it difficult to maintain one’s daily life, live independently, and perform social roles, significantly reducing individuals’ quality of life [4]. In addition, unlike general diseases, dementia cannot be cured to allow patients to return to their previous lives but instead results in a loss of cognitive function and the need for continuous care [5]. Therefore, dementia is a disease for which care must be based on not only knowledge of pathological processes or treatment methods but also psychological and emotional understanding of patients with dementia. Thus, person-centered care has been emphasized recently.

Person-centered care refers to providing individualized nursing care to patients with a common meaning of human-centeredness. Specifically, person-centered care refers to the perception and practice of caregivers who not only meet patients’ psychological needs but also respect their values, abilities, and desire to maintain autonomy, self-esteem, and independence [6]. Thus, person-centered care is a system that emphasizes the need to understand each person’s characteristics and respect and reflect their preferences [7]. It requires more than simply providing patients with what they want, as caregivers must also communicate smoothly with them to find solutions considering the individual’s lifestyle and social situation, putting the patient and their family first in all decisions [8]. Person-centered care for patients with dementia is essential because everyone requires individualized nursing care to maintain their physical and mental health as much as possible. Furthermore, families should also be included as targets of care. Person-centered care can positively affect not only the care recipients but also the nurses who provide care, indicating the need for multifaceted research on this topic.

Nurses are the main healthcare professionals who care for patients with dementia, and the demand for nurses is increasing owing to the increase in the number of older adults with dementia. Nursing students will eventually be required to provide nursing services by meeting with dementia patients and their caregivers directly in healthcare settings. Therefore, nursing students must have a correct awareness and positive attitude toward dementia. Going beyond understanding the mind to reach a certain level of empathy is crucial [9]. Empathy is a cognitive element that recognizes the emotions, motivations, and positions of others, allowing one to feel what others do in a similar way [10]. Empathy facilitates therapeutic relationships between nurses and patients and is helpful in providing individualized nursing care. Furthermore, empathy can influence a patient’s disease course and outcome, indicating its role as a major influencing factor in person-centered care [9]. Therefore, this study aimed to determine the extent of the influence empathy has as a critical variable in nursing students’ person-centered care for patients with dementia.

Despite the increasing emphasis on fostering person-centered care among nursing students, previous Korean studies have primarily focused on practicing nurses working in long-term care hospitals, geriatric settings, or cancer wards. Prior studies mainly examined isolated variables such as empathy, professionalism, work environment, or attitudes toward dementia as independent predictors of person-centered care. For example, empathy has been identified as an important psychological factor associated with therapeutic communication and individualized nursing care, whereas nursing professionalism has been linked to holistic nursing values and patient-centered attitudes. In addition, clinical practice environments and adaptation experiences have been reported to influence nursing students’ professional development and clinical competency.

However, most previous studies examined these variables independently using regression-based approaches rather than integrated structural models. In particular, few studies have simultaneously investigated attitudes toward dementia, clinical practice training environment, clinical practice adaptation, nursing professionalism, and empathy within a single explanatory framework targeting nursing students. Furthermore, limited research has examined how educational, environmental, and psychological factors interact to influence person-centered care competency among nursing students who are expected to provide future dementia care.

Therefore, this study aimed to establish and verify a structural equation model explaining person-centered care competency among Korean nursing students caring for patients with dementia. This study contributes to the existing literature by integrating personal factors, environmental factors, learning experiences, and psychological variables into a single explanatory framework and by identifying both direct and indirect pathways affecting person-centered care.

Social cognitive career theory [11] provided an appropriate theoretical foundation for this study because the theory explains how personal characteristics, environmental conditions, learning experiences, and cognitive factors interact to shape behavioral outcomes and professional development. In the context of nursing education, nursing students’ attitudes toward dementia may influence their adaptation to clinical practice, while clinical practice environments may affect professional identity formation and empathy development. These factors may ultimately be associated with the development of person-centered care competency. Therefore, social cognitive career theory was used to explain the structural relationships among the variables included in this study.

**Figure 1:**
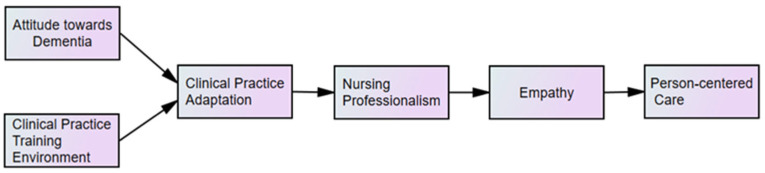
Theoretical framework.

This study established a theoretical framework based on social cognitive career theory [11] to identify the hypothesized relationships and degrees of influence among factors associated with Korean nursing students’ provision of person-centered care to patients with dementia. Social cognitive career theory posits that various personal (e.g., gender, individual characteristics, personality, and status) and environmental factors (e.g., economic means, social support, and role models) interact to affect individual development and cognitive and behavioral attitude formation. Thus, social cognitive career theory explains the process of career development and career choice based on personal, cognitive, and environmental factors, such that the path and influence may vary depending on the factors relevant to the research participants. Therefore, the hypothetical model proposed in this study comprises two exogenous variables, three mediating variables, and one dependent variable based on previous studies to provide a multifaceted and clear explanation. Specifically, attitudes toward dementia were set as an exogenous personal factor, and clinical practice training environments were set as environmental factors. Learning experience was used as the mediating variable set as clinical practice adaptation, and expectation was set as nursing professionalism. Previous studies have reported that empathy is a major influencing factor of person-centered care; therefore, empathy was set as an additional variable and person-centered care as a dependent variable (Figure 1).

## 2. Materials and Methods

### 2.1. Design

This was a covariance structure analysis study to establish a hypothetical model of attitudes toward dementia, clinical practice training environment, clinical practice adaptation, nursing professionalism, empathy, and person-centered care among Korean nursing students. The appropriateness and hypotheses were also verified.

### 2.2. Participants

The participants were nursing students recruited from three nursing colleges located in a metropolitan area of South Korea using convenience sampling. Eligibility criteria included students who had completed at least one semester of clinical practice training and voluntarily agreed to participate in the study. Students who had incomplete questionnaire responses exceeding 10% of the total items were excluded from the final analysis. A total of 313 questionnaires were included in the final analysis. Based on this standard, the acceptable range for the number of samples in this study was 225 to 500, which was an appropriate number of samples for this study [12].

### 2.3. Research Tools

#### 2.3.1. Attitude Toward Dementia

The attitude toward dementia scale developed by O’Connor and McFadde [13] and adapted for use with a Korean population by Choi et al. [14] was used in this study. Participants’ attitudes toward dementia were assessed using 20 items, with scores ranging from 1 to 7. Higher scores indicated a more positive attitude toward dementia. Regarding reliability, Cronbach’s α was 0.85 at the time of development [13] and 0.83 in the present study.

#### 2.3.2. Clinical Practice Training Environment

In this study, the clinical practice training environment was assessed using a tool developed by Dunn [15] and revised and supplemented in Korean by Han [16]. The measure comprises 19 items across five subfactors: three items on the relationship between staff and students, three items on the ward atmosphere, five items on the performance of nursing managers, four items on relationships with patients, and four items on student satisfaction. The scores ranged from 1 to 5, with a higher score indicating a more favorable clinical practice training environment. Cronbach’s ⍺ was 0.84 in Han [16] and 0.82 in this study.

#### 2.3.3. Clinical Practice Adaptation

This study used the tool developed by Park [17] to measure clinical practice adaptation. It includes 12 items and two subfactors: five items on emotional reactions to clinical practice and four items on understanding majors through clinical practice. Scores ranged from 1 to 5, with higher scores indicating greater adaptation to clinical practice. Cronbach’s ⍺ was 0.73 in Park [17] and 0.88 in this study.

#### 2.3.4. Nursing Professionalism

Nursing professionalism was assessed using a measure developed by Yeong, Cho, and Park [18] and modified and supplemented by Han, Kim, and Yun [19]. It consists of 18 items and five sub-factors: six items on professional self-concept, five items on social awareness, three items on nursing professionalism, two items on nursing roles, and two items on nursing independence. The scores ranged from 1 to 5, with a higher score indicating a higher level of nursing professionalism. Cronbach’s ⍺ was 0.86 in Han et al. [19] and 0.85 in this study.

#### 2.3.5. Empathy

The Interpersonal Reactivity Index (IRI) developed by Davis [20], as translated into Korean by Kang et al. [21], was used in this study. The reliability and validity of the Korean-version IRI were previously verified. This scale comprises 28 items and four subfactors: seven items on perspective taking, seven items on imagination, seven items on empathic concern, and seven items on personal distress. The scores ranged from 1 to 5, with higher scores indicating higher levels of empathy. Cronbach’s ⍺ was 0.72 in Kang et al. [21] and 0.70 in this study.

#### 2.3.6. Person-Centered Care

The Korean version of the Person-centered Care Assessment Tool (P-CAT) developed by Edvardsson et al. [7] and translated by Tak, You, and Kim [22], which has previously shown good reliability and validity, was used in this study. This P-CAT consists of 13 items and two subfactors: seven items on individualized care and six on organizational and environmental support. Scores range from 1 to 5 points, and higher scores indicate a greater capacity for positive person-centered care capacity. Cronbach’s ⍺ was 0.72 in Tak et al. [22] and 0.81 in this study.

### 2.4. Data Collection and Analysis Methods

From 1 October to 31 December 2024, self-report questionnaires were distributed to 450 Korean nursing students enrolled in nursing colleges located in a metropolitan area for data collection. Among the 382 questionnaires returned, 69 questionnaires were excluded because of incomplete or insufficient responses and missing data exceeding 10% of the total items. Therefore, 313 questionnaires were included in the final analysis. Cases with minimal missing responses were handled using mean substitution after confirming that the proportion of missing data was low.

SPSS 18.0 for Windows and AMOS 5.0 were used to analyze the data. Structural equation modeling analysis was applied to verify convergent and discriminant validity using higher-order factor analysis in the final model analysis. First, SPSS was used to analyze the participants’ demographic characteristics and the validity and reliability of the questionnaire, and exploratory factor analysis was performed to confirm its construct validity. Second, the hypothetical model was used to verify the validity of the factors for constructing a structural equation model of person-centered care. A hypothetical model was developed using confirmatory factor analysis with AMOS 5.0. Third, convergent validity was used to verify the degree of consistency of multiple scales measuring the same concept; factor loading, squared multiple correlations, construct reliability, and average variance extracted (AVE). Discriminant validity was used to verify the extent to which the scales measured each concept differently. Correlation coefficients and values were used. Fourth, the factors in this study were analyzed using high-, first-, and second-order factor analyses. Finally, χ^2^, χ^2^/df (≤3.00), adjusted goodness of fit index (AGFI ≥ 0.90), goodness of fit index (GFI ≥ 0.90), comparative fit index (CFI ≥ 0.90), normed fit index (NFI ≥ 0.90), and root mean square error of approximation (RMSEA ≤ 0.10) were used to verify model fit. Bootstrapping with bias-corrected confidence intervals was used to verify the significance of indirect effects. The values of NFI and CFI, which are relative fit indices, were considered to indicate good fit if they were 0.90 or higher. RMSEA, which considers the model’s parsimony of the model, indicates a very good fit when <0.05, a good fit when <0.08, a fair fit when <0.10, and a poor fit when >0.10 [12].

## 3. Results

### 3.1. General Participant Characteristics

To determine participants’ general characteristics, data were collected on gender, age, grade, religion, personality, and satisfaction with major as well as whether they had taken courses related to dementia, participated in dementia-related training, had experience with social service programs related to older adults, and had a family history of dementia. Participants’ experiences caring for patients with dementia were also investigated and analyzed.

The participants comprised 266 (85%) female and 47 (15%) male students, and their average age was 23.75 years. The sample included 184 third-year students (58.8%) and 129 fourth-year students (41.2%), and the majority reported they were not religious (206, 65.8%). Regarding personality traits, 150 (47.9%) participants had mixed traits, 107 (34.2%) were introverted, and 56 (17.9%) were extroverted. Furthermore, 249 (85.9%) participants had taken courses related to dementia, whereas 164 (52.4%) had no experience with dementia-related training. A total of 193 (61.7%) participants had experience with social service programs for older adults, 52 (16.6%) had a family history of dementia, and 98 (31.3%) had experience caring for patients with dementia.

### 3.2. Correlations Among Variables and Validity

The fit of the initial model with variables included was modified by verifying the convergent validity by removing measurement items and factors with low validity based on confirmatory factor analysis. Discriminant validity was verified to determine whether the factors each measured separate concepts.

For convergent validity, items with standardized factor loadings (λ) less than 0.6 and significance (t) less than 1.96 were removed through the first confirmatory factor analysis. Specifically, one empathy item and one clinical practice adaptation item with standardized factor loadings below 0.60 were removed to improve convergent validity. These modifications did not alter the conceptual structure of the original instruments because the remaining subfactors were retained. After removing these items, the construct reliability values remained above 0.70, indicating acceptable convergent validity, and the final model was used for analysis.

**Figure 2:**
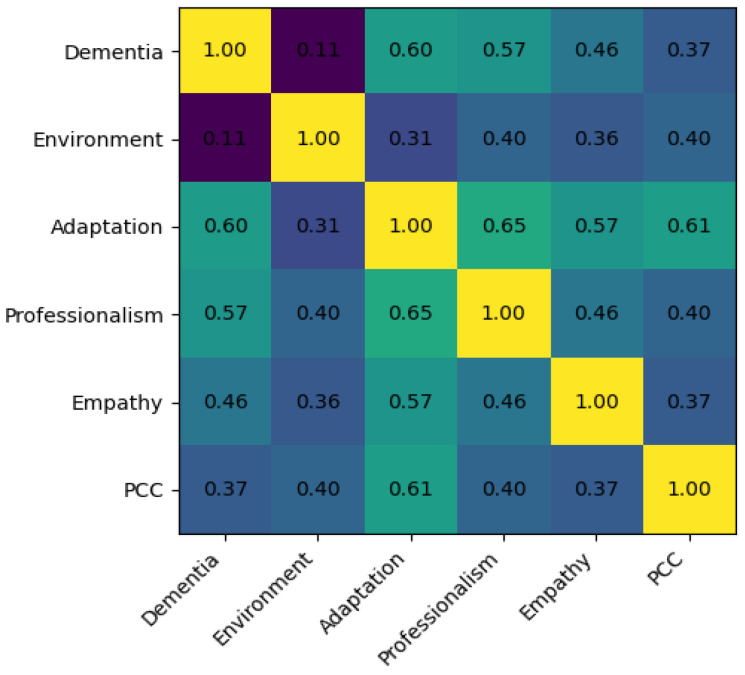
Correlation Plot.

Correlation coefficients and AVE values were used to verify discriminant validity. The correlation coefficients between each factor should be less than the square root of the value. The discriminant validity results verified that the correlation coefficients of all factors ranged from 0.11 to 0.61, which was smaller than the √AVE range of 0.61 to 0.91, indicating good discriminant validity (Table 1, Figure 2). After refinement of the measurement model, most construct validity indices demonstrated acceptable levels. Although the AVE value for person-centered care was slightly below the recommended threshold, the construct reliability exceeded the acceptable criterion and the overall model fit indices supported the adequacy of the measurement model.

### 3.3. Validity Verification and Modification

#### 3.3.1. Test of Hypothetical Model

Based on the results of a preliminary study, 14 hypotheses were proposed, with the exogenous variables of attitude toward dementia, clinical practice training environment, and clinical practice adaptation as mediating variables of nursing professionalism and empathy and person-centered care as the outcome variable. The model fit indices of the research model were as follows: χ^2^/df = 1.83 (*p* < 0.001), GFI = 0.91, AGFI = 0.88, NFI = 0.91, CFI = 0.90, RMR = 0.04, and RMSEA = 0.05. Although the AGFI value was slightly below the recommended threshold of 0.90, the overall model fit was considered acceptable because the remaining fit indices satisfied the recommended criteria. Therefore, the research model was deemed suitable for the sample data, and an analysis was conducted. Based on the structural equation modeling results, 9 of the 14 proposed hypotheses were statistically supported (Table 2).

**Hypothesis** **1.** *“Attitude toward dementia will positively affect clinical practice adaptation” was supported by a path coefficient of 0.58 (CR = 8.12, p < 0.001).*

**Hypothesis** **2.** *“Clinical practice training environment will positively affect clinical practice adaptation” was supported by a path coefficient of 0.25 (CR = 4.30, p < 0.001).*

**Hypothesis** **3.** *“Attitude toward dementia will positively affect nursing professionalism” was supported by a path coefficient of 0.36 (CR = 4.65, p = 0.007).*

**Hypothesis** **4.** *“Clinical practice training environment will positively affect nursing professionalism” was supported by a path coefficient of 0.27 (CR = 4.51, p < 0.001).*

**Hypothesis** **5.** *“Clinical practice adaptation will positively affect nursing professionalism” was supported by a path coefficient of 0.29 (CR = 3.82, p < 0.001).*

**Hypothesis** **6.** *“Attitude toward dementia will positively affect empathy” was not supported by a path coefficient of 0.06 (CR = 0.70, p = 0.059).*

**Hypothesis** **7.** *“Clinical practice training environment will positively affect empathy” was not supported by a path coefficient of 0.10 (CR = 1.51, p = 0.115).*

**Hypothesis** **8.** *“Clinical practice adaptation will positively affect empathy” was supported by a path coefficient of 0.24 (CR = 2.81, p = 0.005).*

**Hypothesis** **9.** *“Nursing professionalism will positively affect empathy” was supported by a path coefficient of 0.44 (CR = 4.83, p < 0.001).*

**Hypothesis** **10.** *“Attitude toward dementia will positively affect person-centered care” was not supported by a path coefficient of 0.02 (CR = 0.24, p = 0.810).*

**Hypothesis** **11.** *“Clinical practice training environment will positively affect person-centered care” was not supported by a path coefficient of 0.07 (CR = 1.09, p = 0.274).*

**Hypothesis** **12.** *“Clinical practice adaptation will positively affect person-centered care” was not supported by a path coefficient of −0.02 (CR = −0.25, p = 0.805).*

**Hypothesis** **13.** *“Nursing professionalism will positively affect person-centered care” was supported by a path coefficient of 0.45 (CR = 4.19, p = 0.024).*

**Hypothesis** **14.** *“Empathy will positively affect person-centered care” was supported by a path coefficient of 0.21 (CR = 2.10, p = 0.036).*

#### 3.3.2. Analysis of the Model’s Direct, Indirect, and Total Effects

Structural equation modeling has the advantage of being able to derive direct, indirect, and total effects easily among variables. The bootstrapping method was used to determine the significance of the indirect effect, and the results are as follows (Table 3). First, when examining the factors affecting clinical practice adaptation, attitude toward dementia (γ = 0.58, *p* < 0.001) and clinical practice training environment (γ = 0.25 *p* < 0.001) were significantly associated with clinical practice adaptation. The explanatory power of these factors was 43%. Second, clinical practice adaptation (γ = 0.29, *p* < 0.001) was significantly associated with nursing professionalism. Attitude toward dementia (γ = 0.17, *p* = 0.004) and clinical practice training environment (γ = 0.07, *p* = 0.003) had indirect effects on nursing professionalism through clinical practice adaptation as a complete mediator. The direct effect of clinical practice adaptation and indirect effects of attitude toward dementia and the clinical practice training environment explained 49% of the variance in nursing professionalism. Third, when examining the factors affecting empathy, nursing professionalism (γ = 0.44, *p* < 0.001) had a direct effect, explaining 48% of the variance. Fourth, examining the factors affecting person-centered care, nursing professionalism (γ = 0.45, *p* = 0.024), and empathy (γ = 0.21, *p* = 0.036) showed significant associations, whereas clinical practice adaptation (γ = 0.21, *p* = 0.013) and nursing professionalism (γ = 0.08, *p* = 0.004) had indirect effects. These factors can explain 40% of the variance in person-centered care.

## 4. Discussion

Person-centered care is considered a competency nursing students must develop during their undergraduate studies and is important for providing high-quality nursing care to patients in actual clinical settings. Therefore, this study examined the factors affecting person-centered care for patients with dementia using a sample of domestic nursing students. The findings verified the fit between the hypothetical model of person-centered care for Korean nursing students and actual data. Accordingly, the effects of nursing students’ attitudes toward dementia and clinical practice training environments on person-centered care through clinical practice adaptation, nursing professionalism, and empathy are discussed below.

First, nursing students’ attitudes toward dementia and the clinical practice training environment had direct positive effects on clinical practice adaptation. No previous research has analyzed this specifically, making it difficult to compare the results of this study with the current literature. However, a previous study conducted with a sample of male nursing students in Korea reported that clinical practice adaptation varies depending on attitude [23]. Furthermore, the clinical practice training environment has been shown to affect clinical practice stress and anxiety [24,25]. Therefore, it can be assumed that nursing students’ attitudes toward dementia and the clinical practice training environment can affect their clinical practice adaptation. Attitudes toward dementia are cognitive attitudes that perceive dementia and patients with dementia either negatively or positively and are the basis for behavioral attitudes when working with patients with dementia [26,27]. A previous study reported that nurses’ positive attitudes toward dementia can improve their empathy in understanding patients with dementia and increase the quality of nursing services [27]. Thus, a positive attitude toward dementia can also affect Korean nursing students’ adaptation to clinical practice. The clinical practice of Korean nursing students is designated as hospital-level medical institutions with more than 300 beds, specialized hospitals such as children’s hospitals, geriatric hospitals, women’s hospitals, integrated nursing and care service operation wards, public health centers, public health subcenters, and community institutions such as industrial sites. To improve clinical practice adaptation, nursing schools and clinical institutions should provide supportive learning environments and systematic pre-clinical preparation programs, including orientation and simulation-based education. These efforts may facilitate students’ successful adjustment to clinical practice settings. Nursing students’ knowledge, skills, attitudes, beliefs, values, ethical standards, and nursing environments affect their adaptation to clinical practice, and these factors are shaped as part of their behavior when preparing to become a nurse [25]. Therefore, a positive attitude and qualitative improvements in the clinical practice training environment are important for nursing students’ active adaptation to clinical practice.

Second, the clinical practice training environment and clinical practice adaptation showed significant associations on nursing professionalism and attitudes toward dementia, while the clinical practice training environment had an indirect positive effect on nursing professionalism through clinical practice adaptation. These results are similar to those reported in previous studies conducted among nursing students and nurses [28,29]. Nursing professionalism is formed and gradually developed through major theories and practical education from nursing students and continues to change after becoming a nurse under the influence of various environmental factors [30]. Practical education has shown a particularly strong effect on nursing students’ nursing professionalism and facilitates the formation of positive nursing professionalism as they adapt to clinical practice [29]. In this study, nursing professionalism was directly and indirectly affected by clinical practice training environment and adaptation. These findings suggest that supportive clinical learning environments and successful adaptation to clinical practice are important for developing nursing professionalism. Therefore, collaborative efforts between nursing schools and clinical institutions are needed to create educational experiences that facilitate professional identity formation among nursing students. A continuous exchange of opinions and agreements between clinical practice institutions and schools should be provided to improve the clinical practice training environment. Schools should secure appropriate clinical practice institutions for each clinical practice participant. Furthermore, clinical practice institutions should understand nursing students’ lack of knowledge and skills, recognize that clinical practice is part of the education students should receive as part of the curriculum, and consider it necessary to help students adapt to clinical practice institutions. An improved clinical practice training environment and better adaptation may contribute to the development of positive nursing professionalism.

Third, nursing professionalism was significantly associated with empathy. These results are similar to those of previous studies conducted among nursing students [31,32]. Empathy is an emotional response that considers others’ situations more than one’s own, and empathy in nursing is a basic prerequisite for providing high-quality nursing and includes behavioral empathy beyond internal empathy [33]. Therefore, patients can only be approached the patient when nurses are providing empathy-based interventions. When nursing is driven by an altruistic motivation to understand and alleviate patients’ difficulties, person-centered nursing based on empathy can be provided. According to previous studies, empathy can be improved through nursing education, and learning strategies regarding care should be included in a healthcare environment that increasingly emphasizes technology [34]. Therefore, nursing education programs should incorporate experiential and reflective learning strategies that promote empathy toward patients with dementia beyond knowledge-based instruction alone.

Fourth, nursing professionalism and empathy showed direct positive associations with person-centered care, whereas clinical practice adaptation had an indirect effect through mediating variables. These findings are partially consistent with previous studies [27,35]. Previous studies have suggested that organizational and psychological factors may influence person-centered care among nurses and nursing students. In particular, nursing professionalism reflects holistic nursing values and professional identity formation, which may facilitate individualized and patient-centered nursing approaches. In addition, empathy enables nurses to understand patients’ emotional experiences and provide individualized nursing care based on therapeutic communication. Therefore, fostering nursing professionalism and empathy may be important for improving person-centered care competency among nursing students caring for patients with dementia.

Nursing professionalism was found to have a direct positive effect on person-centered care, while clinical practice adaptation was found to have an indirect positive effect on person-centered care through nursing professionalism and empathy. These results are consistent with those reported in previous research [18]. Nursing professionalism is the core of holistic nursing practice and provides nurses with appropriate beliefs about the essence of nursing, a systematic view of nursing, and a professional understanding of nursing work. Furthermore, since nursing professionalism develops through not only theoretical education during undergraduate courses but also clinical practice and extracurricular activities [36], a multifaceted educational environment must be created that can foster appropriate nursing professionalism. Previous studies have reported that the formation of appropriate nursing professionalism affects nursing students’ person-centered care before the high or low level of nursing professionalism; therefore, both specific and diverse analyses of nursing students’ nursing professionalism need to be performed. These findings highlight the importance of educational strategies that strengthen nursing professionalism and support the development of person-centered care competency among nursing students.

Empathy is an important factor that improves the ability to guess a patient’s behavior, helps assess the patient’s subjective experience, and provides individualized nursing care, resulting in improved patient well-being and quality of care [33]. Previous studies have reported that empathy improves through education [10,37]. Therefore, before nursing students enter the field to care for patients with dementia, education that can improve empathy, a complex and dynamic concept, and core competency is required. This will help nurses recognize the individual needs of patients with dementia, who experience various symptoms such as a decline in cognitive function and communication difficulties, and provide empathetic patient-centered nursing care. However, few studies have investigated factors related to empathy, such as working with patients with dementia and targeting domestic nursing students who have clinical practice experience. Future studies should investigate additional personal and contextual factors associated with empathy and person-centered care and employ longitudinal or multicenter designs to further validate these relationships.

## 5. Conclusions

This study constructed and verified a structural equation model explaining person-centered care competency among Korean nursing students caring for patients with dementia based on social cognitive career theory. The findings showed that nursing professionalism and empathy had direct positive associations with person-centered care, whereas clinical practice adaptation and nursing professionalism had indirect effects through mediating pathways.

These findings suggest that educational strategies aimed at enhancing nursing professionalism, empathy, and adaptation to clinical practice may contribute to improving person-centered care competency among nursing students. In particular, multidimensional educational approaches that strengthen professional identity formation, therapeutic communication, and dementia care understanding should be considered in nursing education programs.

This study is meaningful because it provides foundational evidence for developing educational interventions to strengthen person-centered dementia care competency among nursing students in Korea.

This study has several limitations. First, person-centered care competency was assessed using self-report questionnaires rather than objective performance-based measures. Second, because participants were recruited from nursing colleges located in a metropolitan area using convenience sampling, caution is needed when generalizing the findings to all Korean nursing students. Third, limited previous studies have examined empathy and person-centered care among Korean nursing students, restricting direct comparison with prior research. Therefore, future multicenter and longitudinal studies are recommended.

## Figures and Tables

**Table 1:** The Correlation and Discriminant Validity of the Model.

Variables	Attitude Towards Dementia	Clinical Practice Training Environment	Clinical Practice Adaptation	Nursing Professionalism	Empathy	Person-Centered Care
Attitude towards Dementia	0.61					
Clinical Practice Training Environment	0.11 ***	0.82				
Clinical Practice Adaptation	0.60 ***	0.31 ***	0.89			
Nursing Professionalism	0.57 ***	0.40 *	0.60 ***	0.91		
Empathy	0.46 ***	0.36 ***	0.57 *	0.65 ***	0.87	
Person-centered Care	0.37 ***	0.40 ***	0.40 ***	0.61 ***	0.52 ***	0.78
Cronbach’s α	0.83	0.82	0.88	0.85	0.70	0.81
CCR	0.75	0.89	0.94	0.93	0.86	0.86
AVE	0.38	0.68	0.80	0.82	0.75	0.61

AVE = Average Variance Extracted; CCR = Composite Construct Reliability. * *p* < 0.05. *** *p* < 0.001. The shaded section denotes discriminant validity; the nonshaded section denotes correlation. Diagonal values represent the square roots of AVE, whereas off-diagonal values represent correlation coefficients among constructs.

**Table 2:** Fit Indices of the Measurement Model.

Variables	χ^2^/df	*p*	GFI	AGFI	NFI	CFI	RMR	RMSEA
Hypothetical	3.12	<0.001	0.81	0.74	0.72	0.76	0.08	0.10
Model—fit	1.83	<0.001	0.91	0.88	0.91	0.90	0.04	0.05

GFI = Goodness of fit index (≥0.90); AGFI = Adjusted goodness of fit index (≥0.90); NFI = Normed fit index (≥0.90); CFI = Comparative fit index (≥0.90); RMR = Root mean residual (≤0.05); RMSEA = Root mean squared error of approximation (≤0.05).

**Table 3:** Direct, Indirect, and Total Effects of Variables in the Final Model.

Exogenous Variable	Endogenous Variables	Estimate	S.E	C.R (t)	Direct Effect (*p*)	Indirect Effect (*p*)	Total Effect	Hypothesis	SMC (%)
Attitude towards Dementia	Clinical Practice Adaptation	0.38	0.05	8.12	0.58 (<0.001)	-	0.58	Supported	0.43
Clinical Practice Training Environment		0.28	0.07	4.30	0.25 (<0.001)	-	0.25	Supported	
Attitude towards Dementia	Nursing Professionalism	0.20	0.04	4.65	0.36 (0.007)	0.17 (0.004)	0.53	Supported	0.49
Clinical Practice Training Environment		0.25	0.06	4.51	0.27 (<0.001)	0.07 (0.003)	0.34	Supported	
Clinical Practice Adaptation		0.24	0.06	3.82	0.29 (<0.001)	-	0.29	Supported	
Attitude towards Dementia	Empathy	0.04	0.05	0.70	0.06 (0.059)	0.37 (0.510)	0.43	Not Supported	0.48
Clinical Practice Training Environment		0.10	0.07	1.51	0.10 (0.115)	0.21 (0.162)	0.31	Not Supported	
Clinical Practice Adaptation		0.21	0.07	2.81	0.24 (0.005)	0.13 (0.071)	0.37	Supported	
Nursing Professionalism		0.46	0.10	4.83	0.44 (<0.001)	-	0.44	Supported	
Attitude towards Dementia	Person-centered Care	0.01	0.06	0.24	0.02 (0.810)	0.31 (0.055)	0.33	Not Supported	0.40
Clinical Practice Training Environment		0.08	0.07	1.09	0.07 (0.274)	0.21 (0.101)	0.29	Not Supported	
Clinical Practice Adaptation		−0.02	0.08	−0.25	−0.02 (0.805)	0.21 (0.013)	0.18	Not Supported	
Nursing Professionalism		0.53	0.13	4.19	0.45 (0.024)	0.09 (0.004)	0.54	Supported	
Empathy		0.23	0.11	2.10	0.21 (0.036)	-	0.21	Supported	

S.E. = standard error; C.R. = critical ratio; SMC = squared multiple correlation. Direct, indirect, and total effects represent standardized effects. “Supported” indicates that the proposed research hypothesis was statistically supported based on the significance and direction of the path coefficient.

## Data Availability

The data presented in this study are available on request from the corresponding author. The data are not publicly available owing to the information contained that could compromise the privacy of research participants.

## Abbreviations

AVE = Average Variance Extracted; CCR = Composite Construct Reliability; CFI = Comparative Fit Index; GFI = Goodness of Fit Index; AGFI = Adjusted Goodness of Fit Index; NFI = Normed Fit Index; RMSEA = Root Mean Square Error of Approximation; RMR = Root Mean Residual; SEM = Structural Equation Modeling

## References

[1] Kim E.Y., Chang S.O. (2021). Qualitative meta-synthesis of nurses’ caring experience for people with dementia. J. Korean Acad. Fundam. Nurs..

[2] Central Dementia Center (2022). Dementia Status in Korea 2022.

[3] Alzheimer’s Association Alzheimer’s and Dementia Facts and Figures. https://www.alz.org/alzheimers-dementia/facts-figures.

[4] Min H.G., Chang J.K. (2020). Meta-analysis of the effects of dementia prevention program for Korean elderly. Korean J. Gerontol. Soc. Welf..

[5] Kim C.G., Lee Y.H. (2020). Nurses’ moral distress on caring for older adults with dementia. J. Korean Gerontol. Nurs..

[6] Kim S., Tak S.H. (2021). Validity and reliability of the Korean version of person-centered practice inventory. J. Korean Acad. Nurs..

[7] Edvardsson D., Fetherstonhaugh D., Nay R., Gibson S. (2010). Development of PCAT. Int. Psychogeriatr..

[8] Lee M.K., Jung H.M. (2019). Relationship between knowledge of dementia care, attitude toward dementia and person-centered care among nurses in geriatric hospitals. J. East-West Nurs. Res..

[9] Brown E.L., Agronin M.E., Stein J.R. (2020). Interventions to enhance empathy. Res. Gerontol. Nurs..

[10] Mirzaei M.A., Abazari F., Miri S. (2020). The effectiveness of empathy training on the empathy skills of nurses working in intensive care units. J. Res. Nurs..

[11] Lent R.W., Brown S.D., Hackett G. (1994). Social cognitive theory. J. Vocat. Behav..

[12] Han S.S., Lee S.C. (2022). Nursing and Health Statistics Analysis Using SPSS/AMOS.

[13] O’Connor M.L., McFadden S.H. (2010). Development and psychometric validation of the dementia attitudes scale. Int. J. Alzheimer’s Dis..

[14] Choi J.Y., Jeong H., Park J.Y., Kim T.H., Lee D.Y., Lee D.W., Ryu S.H., Kim S.K., Youn J.C., Jhoo J. (2015). Factors associated with the attitudes toward dementia in community caregivers: Results from the nationwide survey on dementia care in Korea. J. Korean Geriatr. Psychiatry.

[15] Dunn S.V. (1995). The development of a clinical learning environment scale. J. Adv. Nurs..

[16] Han J.Y. (2010). Nursing students’ perceptions of clinical learning environment (CLE). J. Korean Data Anal. Soc..

[17] Park S.Y. (2016). Development of College Life Adjustment Instrument for Nursing Students.

[18] Yeong K.Y., Cho I.Y., Park S.J. (2022). Mediation and moderation effects of nursing professionalism between caring efficacy and person centered care competency in nursing students. J. Next-Gener. Converg. Technol. Assoc..

[19] Han S.S., Kim M.H., Yun E.K. (2008). Factors affecting nursing professionalism. J. Korean Acad. Soc. Nurs. Educ..

[20] Davis M.H. (1983). Measuring individual differences in empathy: Evidence for a multidimensional approach. J. Pers. Soc. Psychol..

[21] Kang I., Kee S., Kim S.E., Jeong B., Hwang J.H., Song J.E., Kim J.W. (2009). Reliability and validity of the Korean-version of interpersonal reactivity index. J. Korean Neuropsychiatr. Assoc..

[22] Tak Y.R., You S.Y., Kim J.H. (2015). PCAT validation in Korea. J. Korean Acad. Nurs..

[23] Song C.S., Kim J.J. (2017). Male nursing student adaptation. J. Learn.-Centered Curric. Instr..

[24] Kim S.Y., Shin Y.S. (2018). Factors influencing adaptation on clinical practice in nursing students. J. Korea Acad. Ind. Coop. Soc..

[25] Panda S., Dash M., John J., Rath K., Debata A., Swain D., Mohanty K., Eustace-Cook J. (2021). Challenges in clinical learning. Nurse Educ. Today.

[26] Adewuyi M., Morales K., Lindsey A. (2022). Dementia care learning. Nurse Educ. Pract..

[27] Jang S.H., Shin H.H. (2023). Person-centered care in LTC hospitals. J. Health Inform. Stat..

[28] Kim C.H., Kim J.Y. (2019). Nursing professionalism factors. J. Korean Acad. Soc. Nurs. Educ..

[29] Je N.J., Kim J.S. (2020). The influence of clinical learning environment, clinical practice powerlessness, field practice adaptation, and nursing professionalism on caring efficacy in convergence era. J. Digit. Converg..

[30] Vázquez-Calatayud M., Errasti-Ibarrondo B., Choperena A. (2021). Professional development. Nurse Educ. Pract..

[31] Lee M.H. (2019). Dementia attitude and empathy. J. Korean Gerontol. Nurs..

[32] Jun W.H. (2020). Influence of grateful disposition, experience of incivility on nursing professionalism in nursing students who have experienced clinical practice. J. Learn.-Cent. Curric. Instr..

[33] Wu Y. (2021). Empathy in nurse–patient interaction: A conversation analysis. BMC Nurs..

[34] Schuler M.S., Horowitz J.A. (2020). Nursing students’ attitudes toward and empathy for patients with substance use disorder following mentorship. J. Nurs. Educ..

[35] Jung T.M., Kim K.A. (2022). The influence of nursing professionalism, communication competence and nursing work environment of dedicated COVID-19 hospital nurse on person-centered care. J. Korean Acad. Soc. Home Care Nurs..

[36] Cao H., Song Y., Wu Y., Du Y., He X., Chen Y., Wang Q., Yang H. (2023). Nursing professionalism concept analysis. BMC Nurs..

[37] Cheon H.G. (2023). Nurses’ Empathy for Dementia Residents.

